# Accumulation of Stable Full-Length Circular Group I Intron RNAs during Heat-Shock

**DOI:** 10.3390/molecules21111451

**Published:** 2016-10-31

**Authors:** Kasper L. Andersen, Bertrand Beckert, Benoit Masquida, Steinar D. Johansen, Henrik Nielsen

**Affiliations:** 1 Department of Cellular and Molecular Medicine, The Panum Institute, University of Copenhagen, DK-2200 Copenhagen N, Denmark; kasper.andersen@bric.ku.dk (K.L.A.); beckert@genzentrum.lmu.de (B.B.); 2 Molecular Genetics Genomics Microbiology, Université de Strasbourg, CNRS, UMR 7156, Strasbourg 67081, France; 3 Department of Medical Biology, UiT, The Arctic University of Norway, Tromsø N-9037, Norway; steinar.johansen@uit.no

**Keywords:** group I intron, *Didymium iridis*, circular RNA, horizontal gene transfer, molecular modeling, RNA catalysis

## Abstract

Group I introns in nuclear ribosomal RNA of eukaryotic microorganisms are processed by splicing or circularization. The latter results in formation of full-length circular introns without ligation of the exons and has been proposed to be active in intron mobility. We applied qRT-PCR to estimate the copy number of circular intron RNA from the myxomycete *Didymium iridis*. In exponentially growing amoebae, the circular introns are nuclear and found in 70 copies per cell. During heat-shock, the circular form is up-regulated to more than 500 copies per cell. The intron harbours two ribozymes that have the potential to linearize the circle. To understand the structural features that maintain circle integrity, we performed chemical and enzymatic probing of the splicing ribozyme combined with molecular modeling to arrive at models of the inactive circular form and its active linear counterpart. We show that the two forms have the same overall structure but differ in key parts, including the catalytic core element P7 and the junctions at which reactions take place. These differences explain the relative stability of the circular species, demonstrate how it is prone to react with a target molecule for circle integration and thus supports the notion that the circular form is a biologically significant molecule possibly with a role in intron mobility.

## 1. Introduction

Circular RNA species are found dispersedly in biological systems. Compared to their linear counterparts, they have several features that could convey them new biological functions. First, circularity offers resistance towards exonucleases. Concordantly, an increased resistance of circular RNA to degradation has been observed in several cell types [1,2] and the infectious units of highly mobile RNAs such as viroids and satellite RNAs have been found to be circular [3,4]. Second, the joining of the ends creates a unique sequence. This sequence could be used in preferential association of the circular form with protein factors or complementary RNA molecules. Third, end joining by a 2′, 5′ linkage could create a structural alteration that could serve as a recognition motif. End-joining by a 2′, 5′ linkage has been observed in group II introns [5] and viroids [6]. In recent years, circular RNA has emerged as a prominent class of non-coding RNAs in eukaryotes. The circular species are joined by 3′, 5′ linkage and produced by backsplicing. They are widely expressed in a tissue- or developmental specific fashion and plays important roles in sequestering of miRNA or RNA binding proteins thereby regulating gene expression (for recent reviews, see [7,8,9,10]).

**Figure 1:**
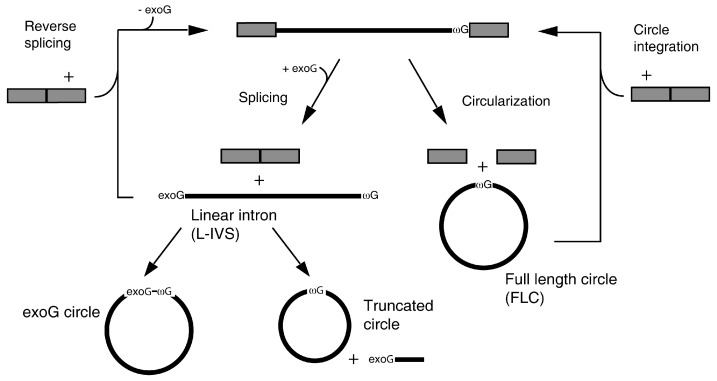
Processing of group I introns. The splicing pathway (left part) is initiated by attack at the 5′ splice site by an exogenous guanosine cofactor (exoG). By two consecutive transesterification reactions the intron splices out of the precursor RNA resulting in ligated exons (grey boxes) and a free linear intron (L-IVS) with the exogenous guanosine (exoG) coupled to the 5′ end. In some introns, attack of the 3′ terminal guanosine (ωG) at an internal site in the L-IVS results in formation of a truncated circular intron RNA and release of a small 5′ end fragment. Alternatively, the attack takes place at the three phosphates of the exoG leading to formation of a circular RNA that incorporates the guanosine cofactor and release of pyrophosphate. The L-IVS can reverse splice into a cognate site as shown, or alternatively, into a new sequence context (intron transposition). The circularization pathway (right part) is initiated by hydrolysis at the 3′ splice site followed by an attack of the ωG at the 5′ splice site. This produces a full-length intron circle (FLC) and un-ligated exons. The FLC can integrate into a target RNA.

Self-splicing group I introns (for reviews, see [11,12]) are a particularly rich source of circular RNAs. They are found widespread, but sporadically in bacteria and their phages, chloroplasts, mitochondria, eukaryotic viruses, and in the nuclear rDNA of some eukaryotic microorganisms. Group I introns catalyse their own splicing by two coupled transesterification reactions that result in ligated exons and a linear intron with a guanosine co-factor added to the 5′ end. In addition to this main pathway, three types of circular RNAs have been found as a result of processing of group I introns (Figure 1). The first type is truncated intron circles that results from circularization of the spliced out intron. Here, the intron terminal residue (ωG) makes an attack at an internal phosphodiester bond at a site near the 5′ end and forms circles with concomitant release of a short RNA derived from the 5′ end. The second type is full-length circles (FLC) that form as the main product of the circularization pathway that has been extensively characterized in nuclear group I introns that are inserted into the rRNA genes [13]. This is an alternative pathway to splicing initiated by hydrolysis at the 3′ SS followed by attack of ωG at the 5′ SS. The products are FLC and un-ligated exons. Finally, circles that are one nucleotide longer than full-length were described in an in vitro study of splicing of the *Anabaena* tRNA^Leu^ intron [14]. These circles are formed by ωG attack at the triphosphate of the GTP that was coupled to the intron 5′ end during the first step of splicing. Thus, pyrophosphate is released and the circles incorporate the guanosine co-factor.

The biological significance of group I intron circular RNA is uncertain. Truncated circles from many variants of the *Tetrahymena* rRNA intron have been characterized in vitro [15,16,17] and their presence in vivo has been documented [18]. Here, the turnover of the circles was found to be very rapid, similar to that of the linear form of the spliced out intron. Truncated circular forms have also been found in vitro and in vivo in a number of other group I introns [13]. In contrast to the truncated circles, the FLCs can be considering genomic in the sense that all intronic sequence information is present. They can re-open by hydrolysis or transesterification, and their presence in cellular RNA is well documented [13,19]. Evidence from both in vivo [20] and in vitro studies suggests that the FLC can integrate into target RNA by an unknown mechanism. This would be an alternative to the well-established mobility of group I introns at the DNA level by a homing mechanism [21] and at the RNA level by reverse splicing [20,22]. The third class of circular group I introns incorporate the guanosine co-factor and thus conserve the bonding energy from the first step of splicing. They may be involved in intron mobility by reverse splicing but their presence in vivo has not been documented.

In the present paper, we focus on the structure and expression of the full-length intron circles formed in the circularization pathway. This pathway has been observed in parallel with the splicing pathway in vitro and in vivo for numerous nuclear group I introns [13,19,23]. We have studied the Dir.S956-1 intron from the myxomycete *Didymium iridis* because this intron has a much more complex biology than most other introns. Dir.S956-1 is a twin-ribozyme intron [24,25] composed of a conventional group I splicing ribozyme (DiGIR2) into which is inserted a lariat capping ribozyme (DiGIR1; [26,27]) followed by a homing endonuclease gene (HEG). The splicing ribozyme is entirely responsible for splicing out the intron as well as all steps of the circularization pathway. In vitro experiments have demonstrated that processing of Dir.S956-1 is equally partitioned between the two pathways [24,25]. In vivo observations suggest the two pathways to be competing in growing amoebae and flagellates [24,25]. Although the partitioning between the two pathways has so far not been directly addressed in the in vivo situation, it is assumed that splicing is by far the dominant pathway.

We have used quantitative RT-PCR (qRT-PCR) to investigate the copy number of FLC in amoebae and flagellates. We estimate that circularization pathway products constitute 1% of total Dir.S956-1 intron processing products in exponentially growing cells and find that the resulting FLC is predominantly, if not exclusively, nuclear. We show that the copy number of FLC as well as the proportion of primary transcripts undergoing circularization can be influenced by external factors, such as heat-shock. The relative stability of the FLC is surprising in view of its inclusion of two ribozymes that both would be expected to linearize the circle. Structure probing analysis and molecular modeling of the linear and the circular forms of the intron reveals that this is primarily due to relaxation of the active site in the circular form. We furthermore suggest that peripheral structures play a role in regulation of the two reaction pathways in response to external stimuli. Thus, group I ribozymes, like many other RNAs, may function as molecular sensors.

## 2. Results

### 2.1. The Copy Number of FLC Is Sensitive to External Stimuli

**Figure 2:**
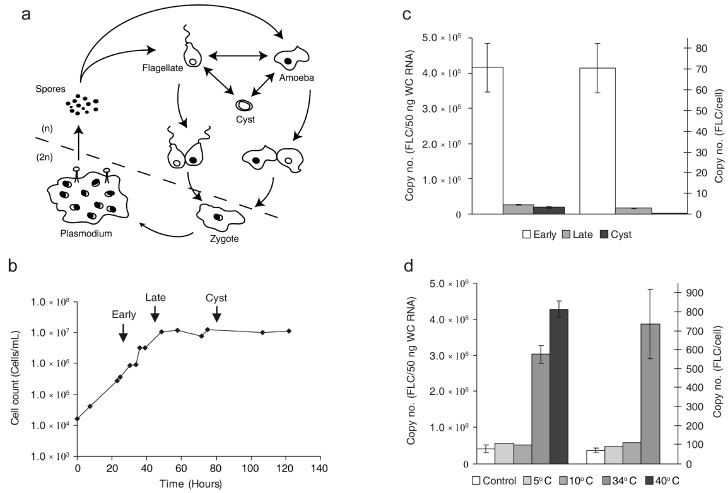
(**a**) The *Didymium iridis* life cycle is divided into microscopic haploid stages (n) and a macroscopic diploid stage (2n). The haploid cell can reversibly transform between amoeba, flagellated cell and cyst depending on environmental factors. Two compatible flagellates or amoeba fusing to form a diploid zygote initiates sexual reproduction. The zygote grows into a multinucleate plasmodium from which fruiting bodies and spores develop. Spores germinate to form amoebae or flagellates thereby completing the cycle; (**b**) Growth course of *Didymium iridis*. The initial growth after parallel inoculation of *D. iridis* and the *Escherichia coli* food source is exponential. Growing in parallel the *Didymium* cells will gradually clear the suspension of *E. coli* and transform from amoebae to flagellates. Then, the growth rate rapidly decreases and the cells transform into dormant and resistant cysts. The cells can be induced to excyst by addition of a bacterial food source. RNA was harvested at the indicated time points: Early (amoeboid cells), Late (flagellated cells) and Cyst; (**c**) Copy number of FLC per 50 ng whole cell (WC) RNA (left *y*-axis) and per cell (right *y*-axis) in RNA isolated from the three time points; (**d**) Copy number of FLC per 50 ng WC RNA (left *y*-axis) and per cell (right *y*-axis) in exponentially growing cells submitted to cold-shock at 5 °C or 10 °C or heat-shock at 34 °C or 40 °C, respectively. FLC per cell for heat-shock at 40 °C cells was omitted due to impaired cell count. Values in (*c*,*d*) are given as mean ± standard error of the mean.

We hypothesized that if the FLC is a biologically significant molecule, the copy number should respond to environmental conditions. First, we analyzed the FLC copy number during exponential growth and starvation-induced encystment (Figure 2a) known to affect pre-rRNA processing [28]. A typical growth curve of *Didymium iridis* in suspension culture at 25 °C is depicted in Figure 2b. RNA was sampled from exponentially grown cells (mostly amoebae), cells at the transition from exponential growth to stationary phase (mostly flagellates), and cysts. The RNA content of the cells declined during the experiment with 8.3 pg per cell in the exponential phase, 5.5 pg per cell in the transition phase and 0.6 pg per cyst, corresponding to 1:0.66:0.07 ratios. The copy number of FLC also showed a decline during the course of growth (Figure 2c). However, the decline was more dramatic from 70 copies per cell in exponential phase to less than one copy per cyst corresponding to ratios 1:0.04:0.003. This demonstrates that less FLC is being produced or that FLC is specifically degraded during starvation-induced encystment. A fractionation study of exponential cells showed that most, if not all, FLC was located in the nucleus (Supplementary Materials Figure S1).

Another set of conditions that are relevant to myxomycete biology is low and high temperatures although only few reports on this are found in the literature. *Didymium* diploid plasmodium forms a macrocyst if exposed to temperatures of 7 to 10 °C for 18 h or 35 °C for 3.5 h [29]. A study of heat-shock protein induction in *Physarum* demonstrated 32 °C as the highest physiological temperature and 37 °C as the highest non-physiological temperature [30]. Based on this we chose temperatures of 5 °C and 10 °C and will refer to this as cold-shock and 34 °C and 40 °C for heat-shock treatment. It should be emphasized that no molecular study was conducted to justify these terms, but it was evident by visual inspection that the *Didymium* cells were affected by the most extreme temperature regimes. Growth for 1 h at 5 °C resulted in 4% of the cells forming cysts, whereas growth at 10 °C had no apparent effect. During growth for 1 h at 34 °C the cells showed a clear tendency to aggregate, and at 40 °C the aggregation was so pronounced that it was impossible to count the cells. The cellular RNA amount was only slightly affected by growth at low or high temperatures. However, the FLC copy number was significantly higher in cells grown at 34 °C or 40 °C compared to the FLC number in cells grown at the standard 25 °C. In Figure 2d this up-regulation is expressed in relation to amount of input RNA as well as cell count. At 34 °C the up-regulation was 7.3 and 10.4-fold, respectively, and at 40 °C the increase in relation to amount of input RNA is 10.3-fold (at this temperature the cells aggregated and could not be counted). In contrast, cold-shock did not have a significant effect on the steady state level of FLC at either temperature (Figure 2d) even though growth at 5 °C had an effect in inducing encystment. The ratios of FLC in cold-shocked cells compared to control cells were 1.3 and 1.2 relative to RNA amount and 1.3 and 1.6 relative to the cell count (for 5 °C and 10 °C cold-shock respectively). In conclusion, these experiments show that the copy number of FLC can be significantly up-regulated to more than 500 copies per cell in response to an environmental factor, demonstrating that FLC formation is unlikely to simply reflect the level of ribosomal RNA synthesis and processing.

### 2.2. FLC Is Very Similar in Structure to L-IVS But Has an Unstable Active Site

Accumulation of FLC within cells is surprising in view of the rapid turnover of truncated circles in *Tetrahymena* [18] and the fact that FLC maintain ribozyme activity and is prone to undergo conversion to a linear form by hydrolytic cleavage at the circularization junction. To understand the structural reasons for accumulation of FLC we conducted a structure probing analysis of the splicing ribozyme component (DiGIR2) of the twin-ribozyme intron using a similar analysis of the linear form (L-IVS) as a reference. The probes report on different aspects of the structure. Unpaired (in the Watson-Crick base pairing sense) nucleotides are revealed by DMS (A > C), kethoxal (G), DEP (A), CMCT (U >> G), RNase T1 (G), RNase T2 (unspecific), and RNase A (U,C), flexible nucleotides are probed by Pb^2+^ and in-line probing, and base paired segments are elucidated by RNase V1. A DiGIR2 construct in which DiGIR1 and the HEG were removed from the P2 segment of the full-length intron was used for practical reasons. The resulting P2 stem contains no foreign sequence insertions and has a length comparable to that of other group IE introns. The DiGIR2 construct carries out the same reactions and accumulates splicing and circularization products in vitro in the same proportions as the full-length intron [13,25,31]. Since two different structural variants of the same intron (L-IVS and FLC, respectively) are being compared, it was critical to ensure that the correct folds of the molecules were investigated. Rather than relying on renaturation protocols that would be difficult to verify, structure probing was carried out in the reaction mixture and the relevant molecular species subsequently isolated for analysis. For non-cleavage probes, L-IVS and FLC could be separated on gels after probing due to their different migration. For cleavage probes, we isolated cleaved FLC based on their inclusion of the circle junction using a circle junction-specific, biotinylated PNA and streptavidin beads. Furthermore, we applied primer extension with a circle junction-specific primer in some cases.

**Figure 3:**
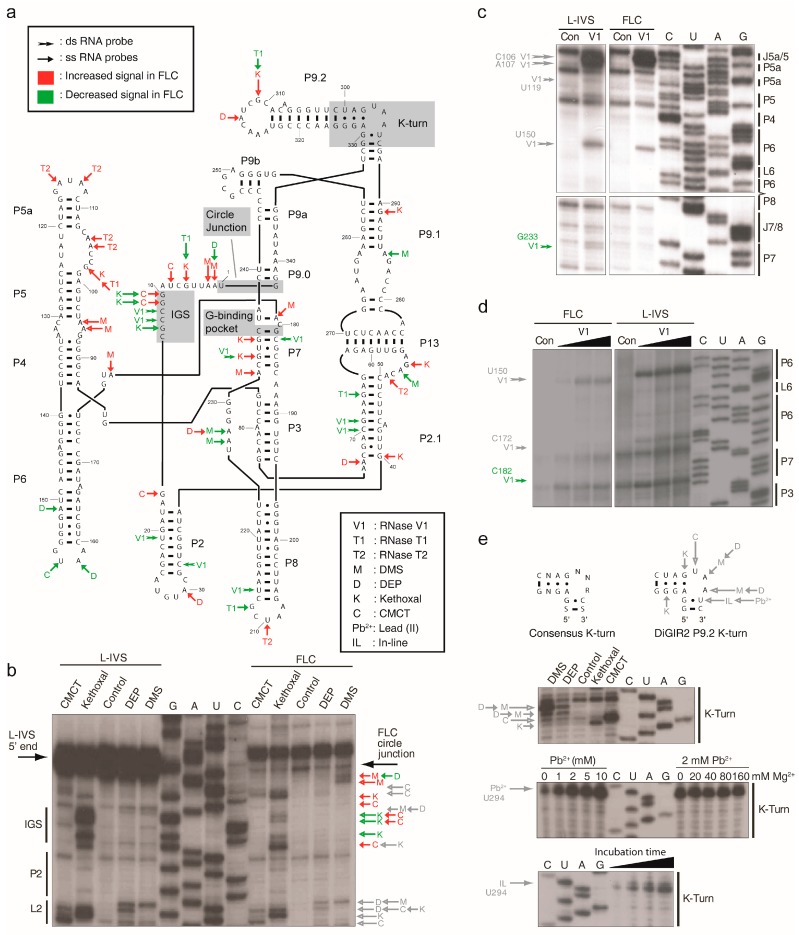
Compilation of probing results for DiGIR2 FLC and L-IVS. Differences in probing signals in FLC compared to L-IVS are highlighted. Red indicates increased signal in FLC, green decreased signal. Grey indicates signals that are similar in the two. The individual structure probing maps of FLC and L-IVS are depicted in Supplementary Materials Figure S2. In addition to the summary secondary structure diagram (**a**), examples of probing gels for the Internal Guide Sequence (IGS) (**b**), P7 comprising essential parts of the G-binding pocket (**c**,**d**), and the K-turn (**e**), are shown. The type of probe is indicated by letters next to the arrows (M: DMS, D: DEP, K: Kethoxal, C: CMCT, T1: RNase T1, T2: RNase T2, V1: RNase V1) and explained in the box in the central part of the figure. The length of the arrows reflects the signal strength. The paired segments are denoted P1–P13, and nucleotide positions within the DiGIR2 construct are indicated by numbers.

Secondary structure diagrams of L-IVS and FLC DiGIR2 based on the general rules for outlining group I introns and displaying the structure probing data are depicted in Supplementary Materials Figure S2. A comparison of the two datasets that highlights the differences between L-IVS and FLC based on 2–5 independent experiments and with insets of examples of primary data is found in Figure 3a. The probing patterns are mostly similar with a few notable differences. First, the FLC differs from the L-IVS in that the exoG is missing and the 5′- and 3′-nucleotides of the intron are covalently linked. The nucleotides on the 5′-side of the junction are accessible to chemical modification at their Watson-Crick edges and thus appear to be solvent exposed (Figure 3b). In contrast, nucleotides 3′ to the junction appear inaccessible. The modification pattern of the circle junction region of the FLC is slightly different from that found within the 5′ end of the L-IVS indicating structural differences (Figure 3b). Several subtle differences were noted in and around the guanosine co-factor binding pocket (G-binding pocket). When ωG is bound into the G-binding pocket located in the narrow groove of P7, it is sandwiched between the last residue of J6/7 and the first residue of P7. Additionally, the other side of the last residue of J6/7 stacks with the 3′ residue of J8/7. In the FLC, these residues are slightly more accessible to Watson-Crick probes than in L-IVS, meaning that the formation of circles is accompanied by the destructuring of the central column of residues interacting in the narrow groove of P7. In further support of this, the V1 cleavages at C182 and G233 in L-IVS were not observed in FLC (Figure 3c,d). Finally, increased accessibility of probes in the upper part of the P4–P6 domain in FLC, in particular at J4/5, J5/5a, and J6/7 suggests a less compact packing of the principal domains and that the docking of the P1 substrate does not occur.

It appears that FLC formation is accompanied by the relaxation of the core of the ribozyme following the second step of the circularization pathway. Once the 5′ exon has been released from DiGIR2, the secondary structure of P1 remains solely based on two A-U pairs that are expected to melt readily and trigger the relaxation process. The unfolding of P1 is expected to destabilize the binding of ωG in its pocket and consequently the 4-nt stack interacting in the narrow groove of P7. Thus, P7 becomes more accessible to the solvent, which implies a greater sensitivity to Watson-Crick probes as compared to L-IVS. The unstructured loop encompassing the P1 residues tethered to ωG cannot dock onto J4/5 and provides the driving force to destabilize the catalytic core by conferring higher dynamics to the nucleotides occupying the narrow groove of P7. Thus, we suggest an induced disorder rather than a different defined position for nucleotides becoming sensitive to Watson-Crick probes specifically in the FLC. As a consequence, these nucleotides have higher dynamics, which indicates an entropy-driven mechanism that would confer them higher positional fluctuations around their average position, which is in good agreement with FLC-specific but faint reactivity and with the observation that reverse splicing/integration can be stimulated by exon mimicking oligonucleotides.

### 2.3. Pb^2+^ Cleavage Sites in the Catalytic Core

**Figure 4:**
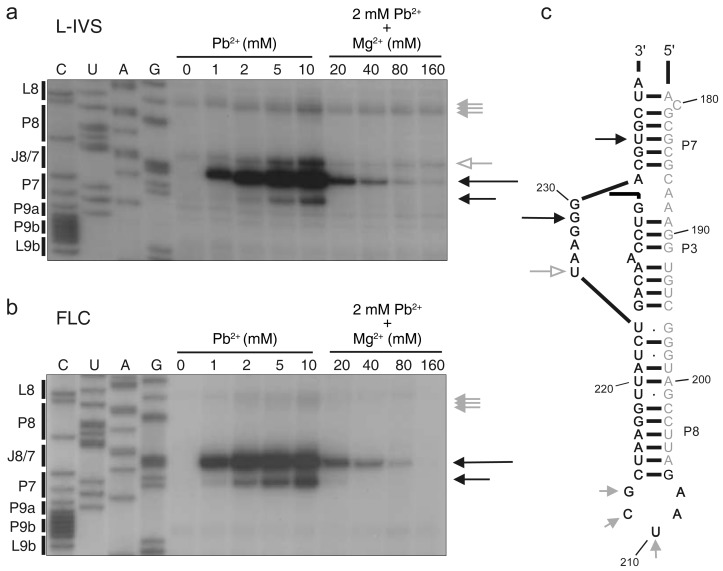
Pb^2+^ induced cleavages in the catalytic core. (**a**) Primer extension analysis of DiGIR2 L-IVS probed with increasing concentrations of Pb^2+^ compared to a control (lane 0–5) and cleavage at a constant Pb^2+^ concentration (2 mM) in presence of increasing concentrations of Mg^2+^ (lane 6–9). To the right, grey arrows indicate Mg^2+^ independent Pb^2+^ cleavage sites; black arrows indicate cleavages affected by Mg^2+^ concentration. The length of the arrows as in Figure 3 represents signal strength (1 to 3). To the left is a DiGIR2 sequencing ladder electrophoresed in parallel with samples and bars indicating structural elements in DiGIR2; (**b**) Primer extension analysis of Pb^2+^ cleavage of DiGIR2 FLC. Figure annotations are identical to (a); (**c**) Results from (a) and (b) depicted on a secondary structure diagram of the J8/7 region. Gray letters indicate nucleotide positions that are not covered in (a) and (b). The open grey arrow indicates the L-IVS specific Pb^2+^ cleavage at U225.

To gain further insight into the structural differences between L-IVS and FLC in P7, we performed a Pb^2+^ probing experiment. Pb^2+^ probing gives information at two levels. Pb^2+^ can displace Mg^2+^ bound at specific binding sites such as the catalytic Mg^2+^ ions bound to the catalytic core in group I introns and cleave the RNA at a nearby phosphodiester bond [32,33]. In addition Pb^2+^ will induce cleavage of the RNA at positions of unconstrained nucleotides. The former type of cleavage site is sensitive to high Mg^2+^ concentrations whereas the latter is not. The results from the Pb^2+^ probing of DiGIR2 L-IVS and FLC core regions are presented in Figure 4. Analysis of the L-IVS shows a prominent signal at G229 (J8/7) and a less prominent signal at U234 (P7). Both of these signals can be competed out by increasing the Mg^2+^ concentration prior to the addition of Pb^2+^. In contrast, a signal at U225 (J8/7) and several minor signals outside the core e.g. in L8 are not affected by increases in Mg^2+^ concentration. This result strongly indicates that Pb^2+^ displaces one or more of the Mg^2+^ ions specifically bound in the catalytic core. The Pb^2+^ probing of FLC is similar with respect to the two signals that could be competed with Mg^2+^ ions but differs in the absence of the signal at U225 (J8/7). This result corroborates the previous finding of differences in the core organization of L-IVS and FLC.

### 2.4. 3D Modeling Supports the Involvement of the P9 Appendages in Circle Formation

**Figure 5:**
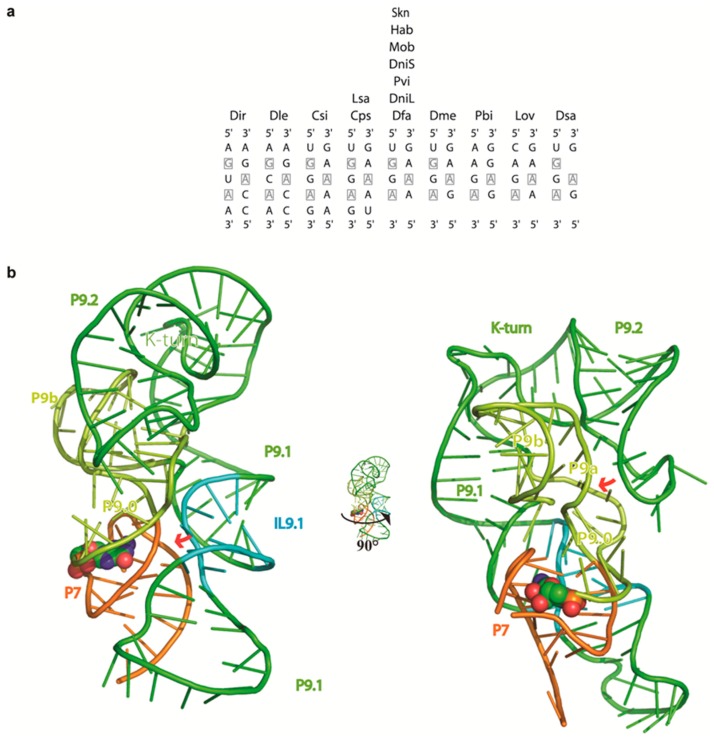
Structural analysis of the P9 domain. (**a**) Sequence structure of the internal loop in P9.1 in 16 sequences of group IE introns. Most sequences show a symmetrical internal loop containing two nucleotides fully conserved and a third one conserved in all but two sequences (Pbi, Lov). These nucleotides (boxed) may be important in the structuring of the loop and/or in its ability to interact with P7. A list of the introns can be found in Supplementary Materials Figure S3; (**b**) Ribbon 3D model showing how the internal loop of P9.1 is thought to be involved in stabilizing P7 (red arrow in left panel) and P9.2 is involved in stabilization of P9.0 (red arrow in right panel), respectively. The internal loop (IL9.1) of P9.1 is depicted in cyan, P7 in orange, P9.0, P9a and P9b in lime green, P9.1 and P9.2 in green. ωG is represented as an atom model with colouring according to atom type (CPK).

In order to better understand the different organization of the core in FLC, we built a 3D model by homology modeling based on available X-ray crystallographic structures of other group I introns. The details of modeling of GIR2 L-IVS, which represents the first whole atom model of a group IE intron, are described in the Supplementary Online Materials that also include presentations of the model of the L-IVS and FLC (Supplementary Materials Figure S4). Of particular interest was the modeling of the P9 appendages that are one of the characteristics of this subgroup. The P9 domain consists of a four-way junction (4 WJ) composed of P9a, P9b, P9.1 and P9.2 that extends the 2-bp P9.0 element stacked onto P7. This allows the ribozyme 3′ residue (ωG) to be accommodated in the G-binding pocket. In order to make the tetraloop L9b interact with its receptor in P5, P9a had to be stacked with P9b in the model. Furthermore, P9.2 was stacked with P9.1 to allow the latter to lie along P7/P3 and interact with L2.1 to finally form the P13 pseudoknot. The conformation of the elements encompassing P2/P2.1 and the P9 insertion is supported by the observation that V1 cleavages are only observed in the P13 strand belonging to P9.1, which is indeed exposed to the solvent in the model whereas the opposite strand is buried. The conformation of the 4 WJ has two major architectural consequences. First, following the formation of P13, the internal loop of P9.1 is in contact with the narrow groove of P7 and thus wraps the catalytic core. Strikingly, a very similar interaction has already been observed, although originating from a P7 insertion instead of a P9 insertion. In the group I intron of the td gene from phage T4 [34] and in the Twort intron crystal structure [35], a loop E motif located in the P7.2 extension stabilizes P7 in a similar way. Three nucleotides in the purine-rich internal loop P9.1 of DiGIR2 are fully conserved in a sequence alignment of group I introns of subgroup IE (Figure 5a) and are thus very likely to directly take part in this interaction. However, the precise arrangement of base pairs taking place in this loop cannot be assessed with certainty due to high variability in the loop size and symmetry. Sequence analysis and structure probing analysis shows that the P9.1 internal loop does not obey the covariation rules and modification patterns observed for the loop E motif [34,36].

The second consequence of the conformation of the 4 WJ is that the predicted K-turn motif bends the P9.2 element by about 120°, which orients L9.2 towards the P9.0/P9a region and allows them to interact (Figure 5b and Supplementary Materials Figure S5b). The sequence of the K-turn in P9.2 strictly fits to the phylogenetic rules specific to this motif [37,38] and the protection pattern corresponds well to the proposed structure with the strongest signals in the two flanking nucleotides A295 and U297 and weaker modification of the central A296 (Figure 3e). In addition, modification signals from two of the guanosines in the non-canonical region of P9.2 were observed in agreement with the formation of the characteristic trans G-A pairs involving their sugar and Hoogsteen edges, respectively (Figure 3a and Supplementary Materials Figure S2). Interestingly, RNases A and T2 did not cleave the K-turn motif. Thus, it seems that RNases cannot recognize the motif as single stranded. The RNase V1 cleavage pattern shows multiple cleavages on both sides of P9.2 (Supplementary Materials Figure S2b) indicating exposure to the solvent. In the model, L9.2 swings in front of J9.0/9a, which becomes buried and thus inaccessible to the solvent as indicated by theoretical accessibility calculations. Such a conformation provides a satisfactory rationale for protections from enzymatic and chemical probes of this FLC region (Supplementary Materials Figure S2b). Moreover, mutational studies point to L9.2 [39] and also J9.0/9a as major actors in the ability of DiGIR2 to naturally carry out the formation of FLC, suggesting that L9.2 may interact with the region encompassing J9.0/9a, although the molecular details of this interaction have not been addressed.

Pb^2+^ cleavage and in-line probing data show different patterns in the P9.2 region of the L-IVS as compared to the FLC (Supplementary Materials Table S2). The 3′ end of L9.2 is unreactive but the 5′ end is strongly cleaved in in-line experiments at position G311 in both RNA species. Moreover, the A307 to A309 nucleotides are significantly less reactive in the FLC than in the L-IVS where this trend propagates up to the K-turn motif. Furthermore, the K-turn motif shows no reactivity at all in the FLC while Pb^2+^ induced cleavages are observed on the 5′-side of the K-turn bulge. These observations are in favor of a greater stability of the P9.2 element in the context of the FLC rather than in the L-IVS, which supports the role of the entire P9.2 appendage as a key element in the mechanism of FLC formation.

## 3. Discussion

### 3.1. The FLC Copy Number Varies in Response to Internal and External Factors

We have used circle specific qRT-PCR to provide the first quantitative data on the FLC corresponding to the product of the circularization pathway in nuclear group I introns. In exponentially growing *Didymium* cells we observed an average copy number of 70 FLC per cell. This is similar to medium abundant mRNA in eukaryotes. The circularization pathway exists in parallel with the splicing pathway in growing amoebae and flagellates. A rough estimate based on qRT-PCR specific for the products of each of the two pathways suggest that 1% of the pre-rRNA precursor is allocated to this pathway (Supplementary Materials Table S1). As the two pathways are equally partitioned during processing in vitro, we suggest that the circularization pathway is specifically suppressed in vivo. A loss of 1% of pre-rRNA precursor could be a marginal cost to the cell. On the other hand, the added cost of having 20 group I introns in the precursor as in *Diderma*, most of which produce FLC, could be substantial [12,24]. The mechanism of suppression is not known, but one possibility is an RNA based switching mechanism that is supported by evidence from structure probing and mutagenesis studies [39]. Alternatively, individual rDNA copies could be dedicated to one or the other pathway based on the sub-nuclear localization of the extrachromosomal rDNA copies.

It has been speculated that the FLC is used in translation of open reading frames located within the intron. Indeed, translation of open reading frames within circular introns is known from Archaea [40]. One argument in favor of circular intron-encoded mRNA is that circularization would offer an alternative stabilization to the m^7^G-cap that is not added because of the nucleolar origin of the mRNA. The observation of the predominantly nuclear localization of the FLC in *Didymium* (Supplementary Materials Figure S1) rules out this possibility and is consistent with our previous finding of a linear and processed form of the I-*Dir*I mRNA on polysomes [41]. The observed nuclear localization is not surprising in view of the fact that FLC contains at least one bona fide nuclear retention signal in the form of a spliceosomal intron.

If the FLC were a biologically significant molecule, the abundance would be expected to vary in relation to cell internal (cell cycle or development) or external (e.g., cellular stress) factors. Encystment and excystment are very frequent transformations that *Didymium* cells undergo repeatedly in their natural environment. We find that the FLC copy number decreases during starvation-induced encystment to below a single copy per cell. We have previously shown that starvation-induced encystment results in accumulation of a 7.5 kb RNA species resulting from cleavage at an intron internal processing site [28] and speculate that this RNA functions as a pre-mRNA for the intron encoded homing endonuclease during excystment. Our finding of a dramatic reduction in FLC during encystment is in accordance with the accumulation of the 7.5 kb RNA because this processing pathway is incompatible with FLC formation. In contrast, we found that growth at elevated temperatures, as an example of cellular stress induced by an external factor, resulted in a 7–10 fold increase in in FLC copy number to more than 500 copies per cell, which is within the range of infectious, circular viroid molecules in infected plant cells [42].

Several mechanisms resulting in FLC accumulation can be envisaged. One of the simplest is a shift in allocation of pre-rRNA from the splicing to the circularization pathway resulting from imbalance of pre-rRNA and ribosomal proteins during cellular stress. Studies from plants and *Drosophila* have shown that pre-rRNA synthesis continues at a slightly reduced level after heat-shock, but that the level of ribosomal proteins and rRNA processing is dramatically reduced [43,44]. If such ribosomal factors were mediating the suppression of the circularization pathway in *Didymium*, heat-shock would relieve the suppression and make the reactions of the intron more similar to what is observed in vitro.

### 3.2. Structural Differences between L-IVS and FLC

The L-IVS and the FLC display similar overall probing patterns, implying only few structural alterations following circularization. Only two nucleotides were found specifically modified by chemicals in the L-IVS compared to 11 positions specific to the FLC. Interestingly, the majority of the differences were clustered in the active site helix P7 and immediate joining segments. In the three crystallized introns from the subgroups IA2, IC1 and IC3 [35,45,46,47] G-binding sites in P7 have been found to be almost identical. The probing results indicate that at least a proportion of the FLC molecules have a relaxed active site with a disassembled G-binding pocket and thus most likely are found in a catalytically inactive conformation following FLC formation. This notion is consistent with the impaired ability of the FLC towards catalyzing re-opening at the circularization junction. Interestingly, a relaxed active site was also reported in an X-ray crystallographic study of the *Azoarcus* group I intron following the second step of splicing [48]. Here, the metal coordination by the exons was lost even though the exons remained bound to the intron through other contacts. Disordered states locally affecting specific RNA regions have been characterized by fluorescence spectroscopy studies of 2-amino purine derivatives of two riboswitches [49,50]. These studies show a shift from a loose to a tight conformation of loops upon ligand binding. It may be a property of many structured RNA molecules that critical parts of the structure adopt a relaxed conformation in the absence of interaction partners or small molecule ligands.

Two high affinity Pb^2+^ cleavage sites were detected in the catalytic core of DiGIR2 in both the FLC and the linear intron. A high affinity Pb^2+^ cleavage site has previously been detected at the second last nucleotide in the J8/7 of the subgroup IC1 *Tetrahymena*, and the subgroup IA2 sunY and the td introns [32]. This Pb^2+^ cleavage site thus reflects a conserved metal ion binding pocket in the intron core of the DiGIR2 intron that can be expected to be present in all group I intron subgroups. In the crystal structure of the *Azoarcus* intron two Mg^2+^ (M1 and M2) were inferred to be coordinating the catalytic center [51]. Five nucleotides in the intron were observed to directly interact with the two ions in the catalytic core and one of these corresponds to the position of the high affinity cleavage site G229 in J8/7 of DiGIR2. This indicates that the M1 ion in the catalytic core is displaced by Pb^2+^ and detected. Additional Pb^2+^ dependent cleavages have been found in P7 and J8/7 of other group I introns [32] suggesting minor group-specific differences in the structure of the active site.

The Pb^2+^ probing pattern also revealed a clear difference between the linear intron and the FLC. A strong cleavage signal at the 5′ end of J8/7 was observed in L-IVS but not in the FLC. This cleavage could not be out-competed by addition of Mg^2+^ ions, and probably reflects a structure of the L-IVS backbone prone to be cleaved by Pb^2+^ ions, resembling the D-loop of the tRNA^phe^ [52]. Indeed, the backbone adopts a gauche^−^/trans conformation around the phosphate group between residues U225-A226 in the L-IVS very similar to the conformation observed between residues D17 and G18 in the tRNA^phe^ [52]. It is worth to note that this specific conformation is due to the recognition of P1 by the 5′ end of J8/7, a situation not found in the FLC due to the destructuring of P1 following the circularization. The observed differences in Pb^2+^ probing between L-IVS and FLC thus similarly reflect differences in the engagement of J8/7 with the remaining P1 sequences. This is consistent with the observation of differences in chemical modifications of this part of the molecule.

The L-IVS and FLC differs in the P1–P2 substrate domain in that L-IVS has the guanosine co-factor attached to its 5′ end whereas the FLC has the 5′ and 3′ ends of the intron covalently linked. The most striking observation is that the 5′ end of the intron is readily accessible to chemical modification in both cases indicating that the Watson-Crick face of the internal guide sequence and the immediate upstream sequence is solvent exposed. This is in accordance with the suggestion that the internal guide sequence in both molecular species promotes reverse splicing/ integration by base-pairing with the target.

### 3.3. The Structure of the P9 Domain Suggests a Role in Promoting Circularization

The P9.2 element in DiGIR2 is particularly interesting when considering the circularization pathway. A time course splicing analysis has shown how a DiGIR2-ΔP9.2 mutant had significantly lower 3′ SS hydrolysis and thus FLC formation, while the splicing reaction was unaffected. The major effect was pinpointed to L9.2 and particularly to the 5′-CAAA sequence on the 3′-side [39]. P9.2 has previously been modeled as the outermost shell secondary structure element with respect to the core of the intron in both the *Tetrahymena* (IC1) [53] and group IE introns [54]. In this way most, if not all, nucleotides of L9.2 would be accessible to modification. The present study shows that the 3′-side of the loop consisting of the sequence that was shown to be important for hydrolysis is protected from modification. Correlated with this, we have verified a previous suggestion that the internal asymmetric loop in P9.2 forms a K-turn motif [39]. The present model shows that optimal stacking at the interface of the four helices forming the 4 WJ between P9a, P9b, P9.1 and the root of P9.2 orients the K-turn so that the tip of P9.2 swings over P9b opening the possibility for the L9.2 loop to interact with the A-rich strand connecting P9a to the ωG carrier P9.0. The potential interaction may block P9.0 on top of P7 to force the docking of ωG into the G-binding pocket and thus promote circularization. This process may be helped by a second interaction between the internal loop of P9.1 and P7 that leads to clamping the G-binding pocket. In this scenario, the L9b/P5 interaction that leads to P1 substrate docking along J4/5 and ultimately to splicing is not needed. Thus, the P9 domain seems to orchestrate the interplay between splicing and circularization by favoring the first step of the latter over the first step of the former. In summary, the P9 domain provides an alternative stabilization mechanism for P7 that re-routes the ribozyme activity towards circularization. Furthermore, since the K-turn can be involved in contacts with protein factors or in the formation of tertiary RNA-RNA interactions [55], it is possible that P9.2 could be a target for molecules produced in the cell according to specific environmental conditions.

### 3.4. A Role for FLC in Intron Mobility?

The Dir.S956-1 intron analyzed in this study is an example of an intron that is mobile within the species (by homing and possibly reverse splicing) [20,56] and between species (as revealed by phylogenetic analysis) (e.g., [19,57]. The FLC has been proposed to be involved in intron mobility by an RNA-based complementary mechanism [31]. Preliminary reports have indeed shown that reverse integration of FLC molecules at the cognate site in ribosomal RNA is feasible [20]. The FLC has several features that would promote such a role. First, it contains all the sequence information of the intron in contrast to the truncated intron circles that are formed by circularization of the spliced out intron. Second, the ends are protected from degradation by exonucleases due to the covalent joining. Third, the FLC retains the potential for catalytic activity as evidenced primarily by its ability to re-open by ligation to the 5′ exon. Fourth, the structure analysis shows that the structure of the P4–P6 and P3–P9 domains of the DiGIR2 structure is conserved upon circularization. However, the P1–P2 domain is structurally perturbed with a solvent exposed internal guide sequence and a G-binding site that appears to be disassembled. This structure ties in with the model of an FLC that is inactivated in hydrolysis but can be re-activated for reverse integration by a slight conformational change perhaps induced by binding to its target RNA. Finally, the copy number of FLC is up-regulated during cellular stress similar to what is seen with many other infectious elements. Thus we conclude that FLC has specific features that are consistent with its proposed role in mobility.

## 4. Materials and Methods

### 4.1. Strains and Sequences

*Didymium iridis* strain Lat3–5 is derived from Pan2–44 isolate, which contains the nuclear rDNA intron Dir.S956-1 [56]. The sequence of the twin-ribozyme group I intron can be found in GenBank (Acc. No. AJ938153).

### 4.2. Growth Conditions

Amoebae were grown in DS/2 medium (1.0 g d-glucose, 0.5 g yeast extract, 0.1 g MgSO_4_, 1.0 g KH_2_PO_4_, 1.5 g K_2_HPO_4_, dH_2_O to 1 L) at 25 °C containing *E. coli* KB as a food source [56]. The cells entered starvation-induced encystment by depletion of the food source. Cells were counted in a Coulter Multisizer (Coulter Electronics Ltd., High Wycombe, England). Cysts were scored by visual inspection after lysis of amoebae and flagellates in 0.5% Nonidet P-40. For heat-shock or cold-shock, exponentially growing amoeba cells at 25 °C were harvested by centrifugation and resuspended in DS/2 medium with *E. coli* KB at 10 °C, 25 °C, or 34 °C, respectively. Aliquots of the cultures incubated at 10 °C or 34 °C were harvested and re-incubated at 5 °C or 40 °C, respectively. Temperature shifts were achieved in 10 min and the cells grown for an additional 1 h before RNA isolation.

### 4.3. RNA Isolation from Cells

Two-ten mL of 10^6^–10^7^ cells/mL was harvested by centrifugation at 450× *g* for 5 min. The pellet was dissolved in 2.5 mL ice-cold RNAzol/10^7^ cells. Following vigorous shaking, 0.2 volume of chloroform was added and the sample left on ice for 20 min. After centrifugation at 1800× *g* for 20 min at 5 °C, the upper phase was isolated, precipitated by addition of 1 volume of isopropanol, and centrifugation at 16,500× *g* for 40 min. The RNA pellet was resuspended and extracted twice with Phenol-Chloroform-Isoamyl Alcohol (PCI, pH 6.6) (BDH), once with chloroform, and precipitated with 3 volumes of 96% ethanol. The RNA concentration was determined with RiboGreen Assay (Molecular Probes, Thermo Fisher Scientific, Waltham, MA, USA) and measured with a Flouroscan Ascent FL using the appropriate software (Thermo Electron Corporation, Waltham, MA, USA). RNA was extracted from each growth condition or cell fraction in 2–5 independent experiments.

### 4.4. Cell Fractionation

Cells were harvested, resuspended in 250 µL ice-cold lysis buffer (20 mM Tris-HCl (pH 8.0), 1.5 mM MgCl_2_, 140 mM KCl, 1.5 mM DTT, 1 mM CaCl_2_, 0.1 mM EDTA, 0.16 mM cycloheximide, 0.5% Nonidet P-40, 500 U/mL RNasin (Promega, Madison, WI, USA; 40 U/µL) and incubated on ice for 5 min. Nuclei were pelleted from the lysate by centrifugation at 10,000× *g* for 10 min at 5 °C. The supernatant containing cytosolic RNA was extracted twice with PCI and once with chloroform and ethanol precipitated. The pellet containing nuclei was dissolved in RNAzol and treated as for WC RNA isolation described above.

### 4.5. Reverse Transcription and Quantitative RT-PCR (qRT-PCR)

50 ng of RNA in 20 µL of RT-buffer (Fermentas, Waltham, MA, USA) containing N_6_ random primers (0.2 µg/µL; GE Healthcare Europe, Brondby, Denmark) was incubated for 1 min at 80 °C (heat denaturation) followed by 5 min at 25 °C (primer annealing). Then, 1 µL of M-MuLV H^−^ reverse transcriptase (200 U/µL; Fermentas) was added and incubation continued for 1 h at 42 °C (cDNA synthesis). For each experiment, a reverse transcriptase minus sample and a 10-fold dilution series of the RNA standard was done in parallel. To each of the samples in the standard dilution series, 50 ng of yeast bulk RNA (Ambion, Thermo Fisher Scientific, Waltham, MA, USA) was added to give same total RNA concentration as the experimental sample. For FLC specific amplification, two primers that would result in a 127 bp PCR-product spanning the circularization junction were selected (C394: 5′-TGATCTTGGGACACGCTACA and C395: 5′-TACTTCCCAACCCAACCAAA). This product was sequenced in several experiments showing only the expected circularization site with no evidence of truncated circles.

A template for making the RNA standard used to calibrate the FLC quantifications was constructed from plasmid pDiSSU1 [31] containing the Dir.S956-1 intron and flanking exons. 1 µg of linearized plasmid was in vitro transcribed and the transcript processed to yield FLC by incubation in reaction buffer (0.5 M KCl, 25 mM MgCl_2_, 40 mM Tris-HCl (pH 7.5), 2 mM spermidine, 5 mM DTT) for 45 min at 45 °C. Then, RT-PCR was applied using a 5′ primer with a T7 promoter sequence (C396: 5′-AATTTAATACGACTCACTATAGGTGTCTGAAAGTAAGGTCTCAAC) and a 3′ primer (C79: 5′-GCCGTTAGGTCGGATGTT) to give a PCR-product of 283 bp that could be transcribed into an RNA representing a part of the FLC including the FLC junction and the primer sites used in qRT-PCR. The RNA standard was treated with Turbo DNase (Ambion), PCI extracted and precipitated. The concentration was determined using a fluorometric assay based on staining with RiboGreen (Molecular Probes) and a 10-fold dilution series ranging from 25 to 2.5 × 10^10^ molecules/µL was prepared.

qRT-PCR was carried out using the LightCycler Instrument (Roche, Basel, Switzerland) and the accompanying software (v.3.5) was used to follow the reaction. The second derivative maximum method was applied to determine threshold cycle and ultimately calculate the FLC copy number. All reagents were from the LightCycler FastStart DNA Master^plus^ SYBR green I kit (Roche). The qRT-PCR parameters were 95 °C, 10 min (1 cycle); 95 °C, 10 s; 60 °C, 5 s; 72 °C, 15 s (40 cycles) followed by a melting curve course 95 °C, 15 s, 65 °C, 15 s, 65 °C to 95 °C (continuous measurement). Each RT-PCR reaction on the various RNA extractions was performed in duplicate.

### 4.6. In Vitro Transcription and RNA Processing

*In vitro* transcription was performed by T7 RNA polymerase (Fermentas) on linearized DiGIR2 plasmid [25] at 5 °C over night to avoid processing of the precursor molecules during transcription. The RNA was uniformly labelled using [α-^32^P]UTP (10 mCi/mL; GE Healthcare Europe, Brondby, Denmark) during transcription. Processing of the RNA (splicing and circularization) was carried out in a buffer that supported structure probing.

### 4.7. Chemical and Enzymatic Structure Probing, RNA Purification and Primer Extension

Chemical and enzymatic probing was performed according to [58]. The chemical probes react preferentially with single-stranded nucleotides (abbreviations and the reactions analysed in parenthesis): dimethyl sulphate (DMS; A > C), kethoxal (KE; G), diethyl pyrocarbonate (DEPC; A), 1-cyclohexyl-3-[2-morpholinoethyl]-carbiodiimide (CMCT; U >> G). The enzymatic probes were (supplier and preference of cleavage in parenthesis): RNase T1 (Sigma, St. Louis, MO, USA; single-stranded G), RNase T2 (Sigma; single-stranded A), RNase A (Ambion; single-stranded U and C), RNase V1 (Ambion; double-stranded RNA without sequence preference). Pb^2+^ probing was carried out according to [59] and in-line probing according to [60]. Chemical probing was carried out in the reaction buffer (70 mM Hepes-KOH (pH 7.8), 10 mM MgCl_2_, 270 mM KCl, 1 mM DTT, 25 μM GTP) and enzymatic probing in the same buffer without DTT. In-line probing was in 50 mM Tris-HCl (pH 8.3), 20 mM MgCl_2_, 100 mM KCl, 0 or 0.2 mM GTP. Pb^2+^-probing was in 25 mM Hepes (pH 7.5), 5 mM MgAc_2_, 100 mM KAc, 0–0.2 mM GTP. All of the cleavage probes (enzymatic, in-line and Pb^2+^) were titrated to single-hit conditions in a linearization assay. In this assay, the linearization of a gel-purified circular RNA to a linear species was assessed by gel electrophoresis.

The RNA was probed directly in the reaction mixture at 45 °C (chemical probes), RT (lead, in-line probing, and RNase V1), or 4 °C (RNases T1 and T2). For the non-cleavage probes, the L-IVS and the FLC were subsequently purified on 5% denaturing (urea) polyacrylamide gels, excised and eluted in 1 mM EDTA, 0.25 mM NaAc (pH 6) followed by analysis by primer extension. Probing that involves cleavage of the RNA rules out the above procedure because the fragments originated from L-IVS and FLC cannot be discerned. In this case, most of the FLC could be analysed by primer extension using a circularization junction specific oligonucleotide. Alternatively, the FLC molecules were purified using a Peptide Nucleic Acid (PNA) probe specific for the junction. In short, the biotinylated probe (PNA 1987: (Biotin)-AGCAATTACCTTTATA-Lys-NH_2_) was annealed to the FLC in the processed and probed mixture of RNA species and the complex subsequently purified on streptavidin-coated magnetic beads. In the analysis of L-IVS after probing with cleavage probes, signals derived from unprocessed precursor molecules could not be discerned from those originating from L-IVS. The results presented are the consensus from two to five independent experiments.

The primers used for primer extension analysis were: C147: 5′-TTGATCGTTGGCCTCA), C339: 5′-ACCTTAGCGATTCTAA), C340: 5′-CCTTTATACCAGCCT) and C151: 5′-CGGCCTAGCAATT ACCTTTATA (junction specific).

### 4.8. Molecular Modeling

The DiGIR2 structure model was built by homology using the program ASSEMBLE, an extended version of MANIP [61] linked to the S2S application [62]. First, eight sequences of representatives of the group IE intron subgroup were aligned using the *Azoarcus* ribozyme as the reference three-dimensional structure [45,63]. Gaps were inserted accordingly in the *Azoarcus* sequence to account for insertions specific to the group IE ribozymes. Following this, ASSEMBLE was used to automatically generate the 3D coordinates of nucleotides from DiGIR2 aligned pair wise to nucleotides from the *Azoarcus* ribozyme. Regions corresponding to insertions (P2, P2.1, P9.1, and P9.2) were built using the interactive functionalities from MANIP implemented in the program Assemble.

## Supplementary Materials

The following are available online at http://www.mdpi.com/1420-3049/21/11/1451/s1, Figure S1: The full length intron circle (FLC) is predominantly confined to the nucleus in exponential growing *D. iridis* cells. Figure S2: Chemical and RNase probing of the linear (L-IVS) and circular (FLC) DiGIR2 intron. Figure S3: Sequence covariation between the P5 receptor and L9 sequence. Figure S4: The FLC and L-IVS DiGIR2 ribozyme 3D model overlay. Table S1: Intron processing pathways. Table S2: Results from Pb2+ and in-line probing of nucleotide 200-318 of DiGIR2 FLC and L-IVS.
