# Supplementary material for: Accumulation of Stable Full-Length Circular Group I Intron RNAs during Heat-Shock
Source: Molecules. 2016 Oct 31;21(11):1451. doi: 10.3390/molecules21111451 (PMC6274462; doi:10.3390/molecules21111451)
Supplement: Supplementary file 1 [file molecules-21-01451-s001.pdf]

Supplementary Materials: Accumulation of Stable Full-Length Circular Group I Intron RNAs during Heat-Shock

Kasper L. Andersen, Bertrand Beckert, Benoit Masquida, Steinar D. Johansen and Henrik Nielsen

Table S1. Intron processing pathways. The ratios of intron full-length circle (FLC) and the intron embedded homing endonuclease mRNA (HEG mRNA) copy numbers determined in parallel by qRT-PCR for the experimental conditions described in the paper. In the lower row, the ratios are normalized to exponentially growing cells at 25 °C. The primers for FLC specific amplification were similar to those described in the main paper. For HEG mRNA specific amplification, the 5′ primer (C397: 5′-GCGTTCTAGGCCCGATG) was designed to span the exon-exon junction created by splicing of the small 51 nt spliceosomal intron found in the HEG of the Dir.S956-1 group I intron. The 51 nt intron is removed as the last step in a series of processing steps leading to the mature mRNA encoding the homing endonuclease and the resulting exon-exon junction sequence that is not present in the precursor or the FLC [1]. Amplification using c397 and a 3′ primer (C398: 5′-CCT TGC TTG TCT GGA TCC TC) resulted in a PCR-product of 126 bp.

Ratio	Early	Late	Cyst	Cold-Shock 5 °C	Cold-Shock 10 °C	Heat-Shock 34 °C	Heat-Shock 40 °C
FLC/HEG mRNA	0.0094	0.0040	0.0077	0.0094	0.0086	0.0214	0.0495
Normalized	1.0	0.4	0.8	1.0	0.9	2.3	5.3

Table S2. Summary of results from Pb^{2+} and in-line probing of nucleotide 200–318 of DiGIR2 FLC and L-IVS. Signal strengths are indicated as "1" (weak), "2" (intermediate), or "3" (strong). "*": no signal; nd: not detected due to proximity to primer. Some successive signals could be due to reverse transcriptase "stuttering". This has only been corrected in the most obvious cases.

Position	Element	Sequence	(1) FLC		(2) L-IVS	
			Pb(2+)	Inline	Pb(2+)	Inline
**200**	P8′	**A**	*	*	*	*
**201**	P8′	**G**	*	*	*	*
**202**	P8′	**C**	*	*	*	*
**203**	P8′	**C**	*	*	*	*
**204**	P8′	**U**	*	*	*	*
**205**	P8′	**U**	*	*	*	*
**206**	P8′	**A**	*	*	*	*
**207**	P8′	**G**	*	*	*	*
**208**	L8	**A**	*	In-1	*	In-1
**209**	L8	**A**	*	In-1	*	In-1
**210**	L8	**U**	Pb-1	In-2	Pb-1	In-2
**211**	L8	**C**	Pb-1	In-2	Pb-1	In-2
**212**	L8	**G**	Pb-1	In-1	Pb-1	In-1
**213**	P8″	**C**	*	*	*	*
**214**	P8″	**U**	*	*	*	*
**215**	P8″	**A**	*	*	*	*
**216**	P8″	**A**	*	*	*	*
**217**	P8″	**G**	*	*	*	*
**218**	P8″	**G**	*	*	*	*
**219**	P8″	**U**	*	*	*	*
**220**	P8″	**U**	*	*	*	*
**221**	P8″	**A**	*	*	*	*

**222**	P8″	**U**	*	*	*	*
**223**	P8″	**C**	*	*	*	*
**224**	P8″	**U**	*	In-1	*	In-1
**225**	J7/8	**U**	*	In-1	Pb-2	In-1
**226**	J7/8	**A**	*	*	*	*
**227**	J7/8	**A**	*	*	*	*
**228**	J7/8	**G**	*	In-1	*	In-2
**229**	J7/8	**G**	Pb-3 (Mg^{2+})	In-2	Pb-3 (Mg^{2+})	In-3
**230**	J7/8	**G**	*	*	*	*
**231**	P7″	**A**	*	*	*	*
**232**	P7″	**C**	*	*	*	*
**233**	P7″	**G**	*	*	*	*
**234**	P7″	**U**	Pb-2 (Mg^{2+})	*	Pb-2 (Mg^{2+})	*
**235**	P7″	**G**	*	*	*	*
**236**	P7″	**C**	*	*	*	*
**237**	P7″	**U**	*	*	*	*
**238**		**A**	*	*	*	*
**239**	P9.0′	**C**	*	*	*	*
**240**	P9.0′	**U**	*	*	*	*
**241**	P9a′	**C**	*	*	*	*
**242**	P9a′	**C**	*	*	*	*
**243**		**C**	*	*	*	*
**244**	P9b′	**C**	*	*	*	*
**245**	P9b′	**C**	*	*	*	*
**246**	P9b′	**C**	*	*	*	*
**247**	L9b	**G**	*	*	*	*
**248**	L9b	**C**	*	*	Pb-1	*
**249**	L9b	**G**	*	In-1	Pb-1	In-1
**250**	L9b	**A**	*	*	*	*
**251**	P9b''	**G**	*	*	*	*
**252**	P9b''	**G**	*	*	*	*
**253**	P9b''	**G**	*	*	*	*
**254**		**U**	*	*	Pb-1	*
**255**		**G**	*	*	*	*
**256**	P9.1′	**U**	*	*	*	*
**257**	P9.1′	**C**	*	*	*	*
**258**	P9.1′	**U**	*	*	*	*
**259**	P9.1′	**G**	*	*	*	*
**260**	P9.1′	**A**	*	*	*	*
**261**	P9.1′	**A**	*	*	*	*
**262**	L9.1′	**A**	*	In-1	*	In-1
**263**	L9.1′	**G**	*	In-1	*	In-1
**264**	L9.1′	**U**	*	*	*	*
**265**	L9.1′	**A**	*	*	*	*
**266**	L9.1′	**A**	*	*	*	*
**267**	L9.1′	**G**	*	*	*	*
**268**	L9.1′	**G**	*	*	*	*
**269**	P13″	**U**	*	*	*	*
**270**	P13″	**C**	*	*	*	*
**271**	P13″	**U**	*	*	*	*
**272**	P13″	**C**	*	*	*	*
**273**	P13″	**A**	*	*	*	*

**274**	P13″	**A**	*	*	*	*
**275**	P13″	**C**	*	*	*	*
**276**	P13″	**C**	*	*	*	*
**277**	L9.1″	**A**	*	*	*	*
**278**	L9.1″	**C**	*	*	*	*
**279**	L9.1″	**C**	*	*	*	*
**280**	L9.1″	**C**	*	*	*	*
**281**	L9.1″	**C**	*	In-1	*	In-1
**282**	L9.1″	**A**	*	*	*	*
**283**	L9.1″	**G**	*	*	*	*
**284**	L9.1″	**A**	*	*	*	*
**285**	P9.1″	**U**	*	*	*	*
**286**	P9.1″	**U**	*	*	*	*
**287**	P9.1″	**C**	*	*	*	*
**288**	P9.1″	**A**	*	*	*	*
**289**	P9.1″	**G**	*	*	*	*
**290**	P9.1″	**A**	*	*	*	*
**291**	P9.2′	**A**	*	*	*	*
**292**	P9.2′	**G**	*	*	*	*
**293**	P9.2′	**C**	*	*	*	*
**294**	P9.2′	**U**	*	In-1	Pb-2	In-1
**295**	(P9.2′)	**A**	*	*	Pb-1	*
**296**	(P9.2′)	**A**	*	*	*	*
**297**	(P9.2′)	**U**	*	*	*	*
**298**	P9.2′	**G**	*	*	*	*
**299**	P9.2′	**A**	*	*	*	*
**300**	P9.2′	**U**	*	*	*	*
**301**	P9.2′	**C**	*	*	*	*
**302**	P9.2′	**U**	*	*	*	*
**303**	P9.2′	**U**	*	*	*	*
**304**	P9.2′	**G**	*	*	*	*
**305**	P9.2′	**G**	*	*	*	*
**306**	P9.2′	**G**	*	*	*	*
**307**	(P9.2′)	**A**	*	*	*	In-2
**308**	P9.2′	**C**	*	*	Pb-1	In-1
**309**	P9.2′	**A**	Pb-1	*	Pb-1	In-1
**310**	L9.2	**C**	Pb-1	In-1	Pb-1	In-1
**311**	L9.2	**G**	Pb-1	In-2	Pb-1	In-3
**312**	L9.2	**C**	Pb-1	*	Pb-1	*
**313**	L9.2	**U**	Pb-1	In-1	Pb-2	In-1
**314**	L9.2	**A**	Pb-1	In-1	Pb-2	In-1
**315**	L9.2	**C**	*	In-1	Pb-1	In-1
**316**	L9.2	**A**	*	*	*	*
**317**	L9.2	**A**	*	nd	nd	nd
**318**	L9.2	**A**	*	nd	nd	nd

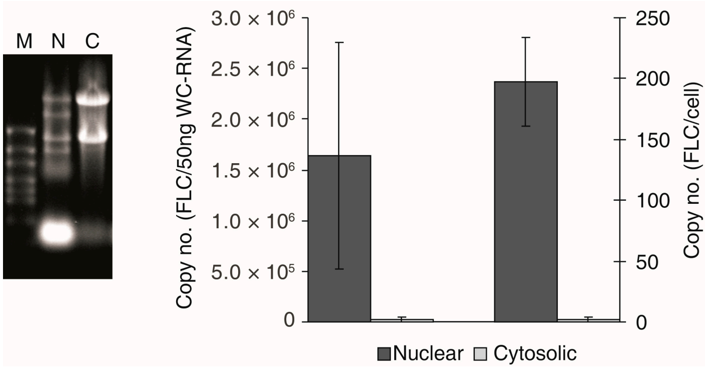

Figure S1. The full-length intron circle (FLC) is predominantly confined to the nucleus in exponential growing *D. iridis* cells. Left panel: 10 μg RNA from cellular fractions used for qRT-PCR detection of FLC was separated on 0.9% denaturing formaldehyde agarose gel and subsequently stained with ethidium bromide. M: Marker, N: nuclear fraction. C: cytosolic fraction. Right panel illustrates the qRT-PCR determined copy number of FLC per 50 ng whole cell RNA (wcRNA) and per cell in nuclear and cytosolic fractions. Values are given as mean ± standard error of the mean (n = 4).

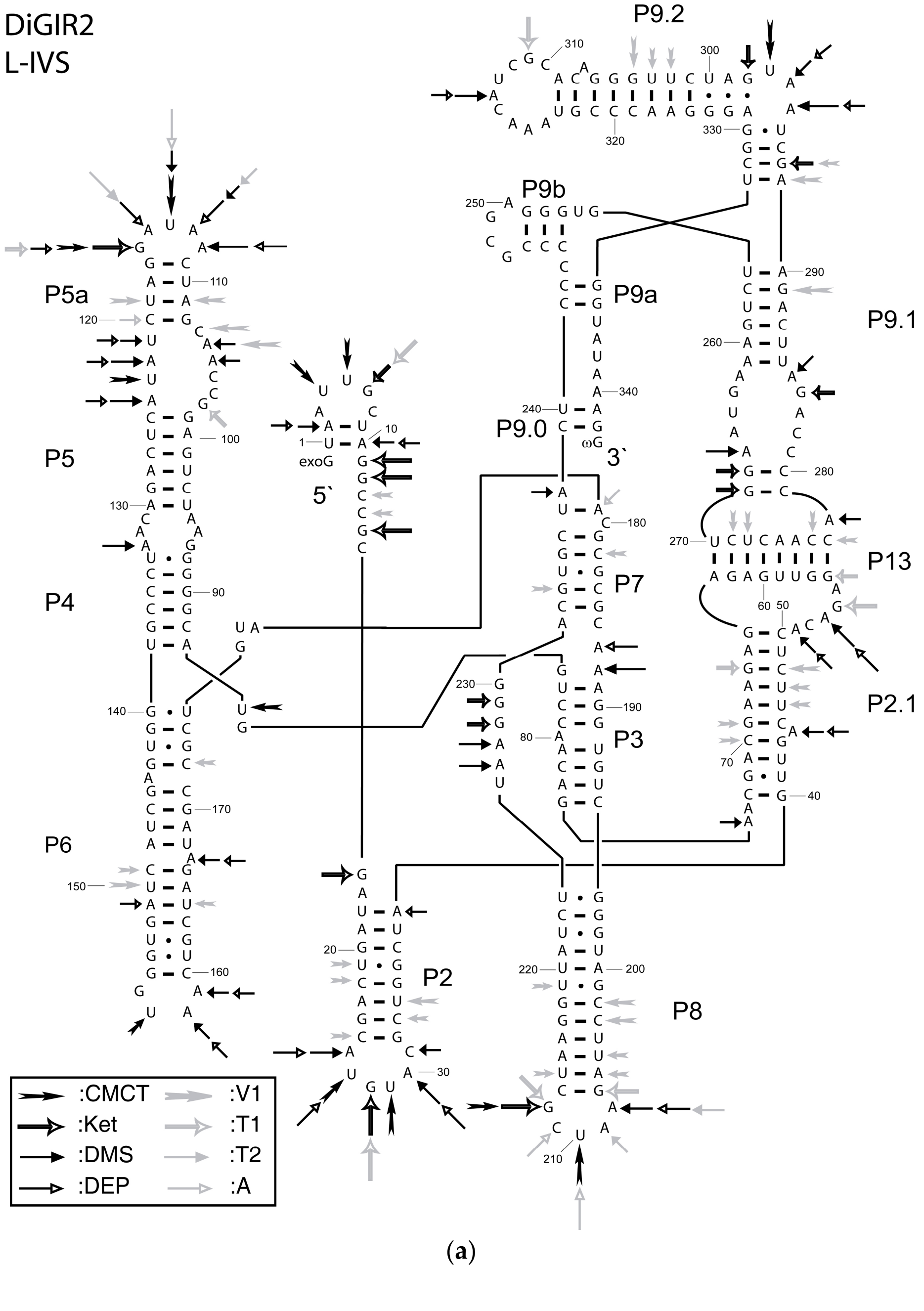

(**a**)

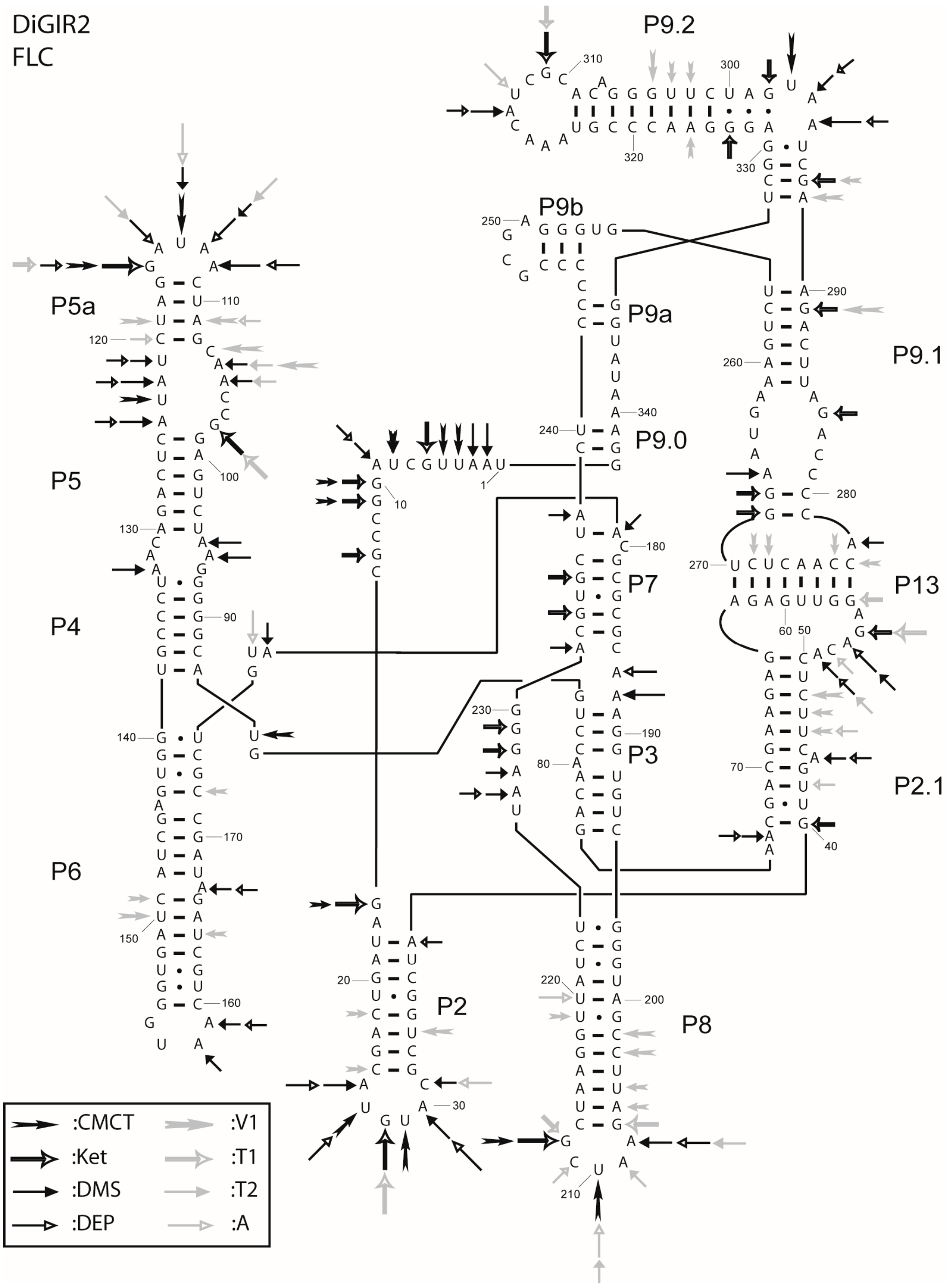
DiGIR2
FLC
P9.2
P9b
P9a
P9.1
P5a
P5
P9.0
P7
P13
P4
P3
P2.1
P6
P2
P8
:CMCT
:Ket
:DMS
:DEP
:V1
:T1
:T2
:A

(**b**)

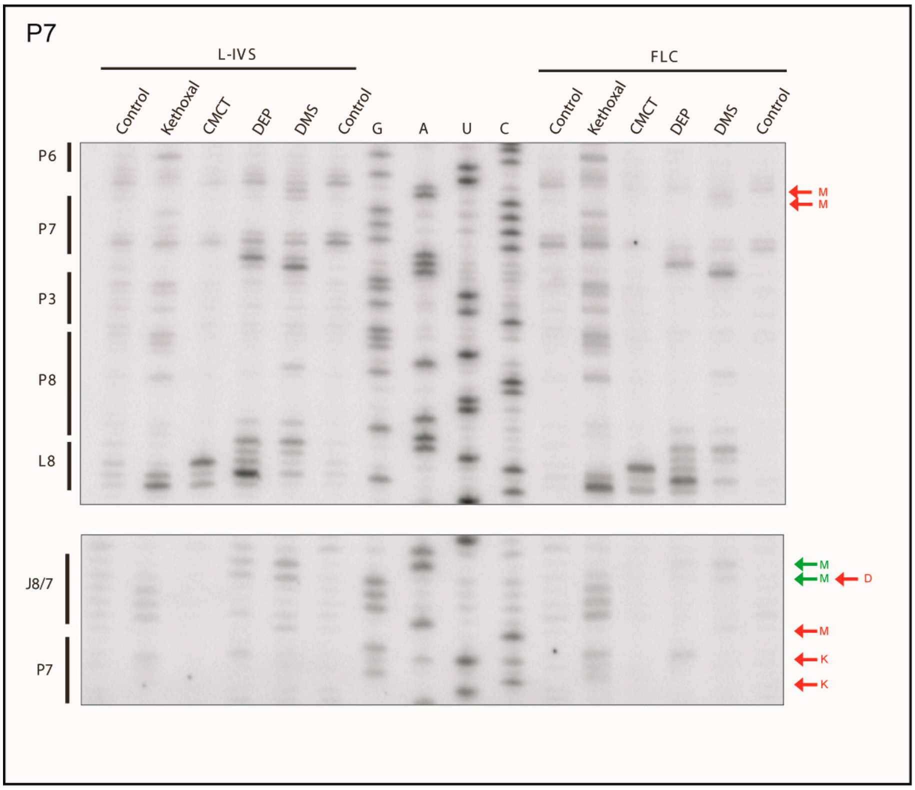

(**c**)

Figure S2. Chemical and RNase probing (**a**) of the linear (L-IVS) and (**b**) FLC DiGIR2 intron. Signals from modifications and cleavages are indicated with arrows on the proposed secondary structure of DiGIR2 L-IVS. The various probes are specified in the inserted box. The strength of modification/cleavage signals has been scored by as 1, 2 or 3; 3 being the strongest. The lengths of the arrows in the figures correspond to these scores. Thus a long arrow indicates a strong signal; (**c**) Sample of chemical probing comprising P7. Red indicates increased signal in FLC, green decreased signal.

1. Description of the Overall Structure of the L-IVS and the FLC

P4–P6 domain. This domain has been shown in some introns to fold early and act as a scaffold in folding of subsequent domains [2]. In DiGIR2, P5 is extended by an asymmetrical internal loop and a helix (P5a). The P5 and P6 helices in L-IVS were confirmed by V1 cleavages and their capping loops were accessible to extensive chemical modification. The central part of the molecule appears to be inaccessible to modification and RNase cleavage. The asymmetrical loop connecting P5 and P5a is readily modified by chemical probes and appears not to contain Watson-Crick base-pairs. Interestingly, two of the strongest V1 cleavage sites overall are found in the single-stranded J5/5a at A106 and C107 indicating stacking of these residues (see Figure S2c). The probing results from FLC were very similar to that of L-IVS, but with some exceptions. An increased accessibility of probes to the 5′ strand of the top of the domain in FLC and a decreased accessibility of probes to the 5′ strand of P6 and parts of L6 indicate a slightly different positioning of this domain in FLC compared to L-IVS, and is most likely linked to the absence of P1 in FLC. Overall, the structure of the P4-P6 domain was confirmed and did not contain major structural alterations in the FLC.

P3–P9 domain. This domain harbours the binding site for the guanosine co-factor and ωG in P7. In DiGIR2, the P9 part is very complex consisting of a 2-bp helix (P9.0) and three hairpins branching from this, some of which have been specifically assigned a role in the circularization pathway [3]. The probing strategy excluded data collection from P9.0″ and P9a″ and the most 3′ part of P9.2 in the L-IVS due to overlap or proximity to the primer used in reverse transcription. Likewise, no data from Pb^{2+}, in-line and RNase probes is available for 16–22 nucleotides spanning the FLC circle junction due to the circular RNA purification method. From analysis of those parts that are

amenable to analysis, P9.0, P9a and P9b appear inaccessible to chemical modification and RNase cleavage, indicating that this part of the structure is buried within the ribozyme. P9.1 and P9.2 are supported by V1 cleavages and the bulge in P9.2 is accessible to chemical modification. In contrast the 9-nt L9.2 and the 10-nt internal loop in P9.1 are only modifiable in a few positions, indicating that they form structured motifs. An 8-nt stretch of L9.1 is cleaved at several positions by V1 and is not modified by chemical probes, indicating an involvement in a long-range base-pairing (P13; see below). P7 and P8 were confirmed by the presence of V1 cleavages, and the joining segments J8/7, J7/3, J7/9.0, and L8 by accessibility to chemical modification. The probing results for FLC were similar to that of L-IVS in P3, P8, and P9. However, several differences were noted in and around P7. The V1 cleavages at C182 and G233 were not observed in FLC. In accordance with this, the FLC was accessible to DMS modification at A179, and kethoxal modification at G233 and G235. In addition, A231 in J8/7 and A178 in J6/7 were modifiable by DMS in FLC, but not in L-IVS. Taken together, the results indicate a structural alteration of the G-binding site in the FLC compared to the L-IVS.

P1–P2 domain. This is frequently referred to as the substrate domain because it harbours the 5′-splice site. In DiGIR2, the P1–P2 domain consists of an 8 base-pair P1 including 6 base-pairs between the internal guide sequence and the 5′-exon, as well as P2 and P2.1 helices. In the DiGIR2 L-IVS, the 5′-exon is removed and the guanosine co-factor is attached to the 5′-end of the intron. L2 is artificial in the sense that DiGIR1 and HEG have been removed by deletion. However, P2 is roughly similar to P2 known from other group IE introns not carrying an insertion. The remainder of P1 is readily accessible to chemical modification, as expected. P2 and P2.1 are confirmed by numerous V1 cleavages and L2 is modifiable with the chemical probes. The major part of L2.1 appears protected against chemical modification consistent with its involvement in a long-range base-pairing interaction with L9.1. In all, the P1–P2 domain appears as the most accessible domain to the probes applied in this study. Obviously, the FLC differs from the L-IVS in that the exoG is missing and the 5′- and 3′-nucleotides of the intron are covalently linked. The nucleotides on the 5′-side of the junction are accessible to chemical modification and thus appear to be solvent exposed. In contrast, nucleotides 3′- to the junction appear inaccessible. The modification pattern of the circle junction region of the FLC is slightly different from that found within the 5′ end of the L-IVS indicating structural differences. The P2–P2.1 part of the domain is mostly similar in the two molecular species.

P13 long-range interaction. This group IE characteristic long-range base-pairing interaction was confirmed by numerous V1 cleavages on the L9.1 strand that consequently is proposed to constitute the surface exposed part of the helix. P13 is found in both the L-IVS and the FLC.

2. 3D Modeling of the Intron (Figure S4)

The model of the core of the L-IVS was built by homology modelling with the *Azoarcus* group I ribozyme crystal structure [4,5]. Using this strategy, all nucleotides equivalent to specific positions in the *Azoarcus* ribozyme from the P3–P9 and the P4–P6 domains were built in accordance with secondary and tertiary interactions that form upon domain assembly. In this context, the precise positioning of the P9 hairpin allowed for the identification of the receptor of the L9 GCGA tetraloop. This receptor is located in the P5 region proximal to the J4/5 internal loop and corresponds to the consecutive base-pairs C97 = G129 and U98–A128 which interact with the sugar edge of A250 and G249 from the tetraloop, respectively. These interactions are in agreement with the covariation usually observed within this tertiary structure motif [6] and are moreover supported by sequence covariation in a set of representatives of the group IE ribozymes (Figure S4). Homology modelling was also applied to build the characteristic GoU base-pair of the P1 substrate helix onto the core. The P1 hairpin was further built in the form appearing in L-IVS before the first step of the circularization pathway. The differences between DiGIR2 and the *Azoarcus* ribozyme cores were addressed by taking advantage of the more closely related *Tetrahymena thermophila* ribozyme crystal structure deprived of P1, P2 and P2.1 [7]. In DiGIR2, P3 is a 7-bp stem with a bulge between the fourth and fifth base-pairs. J8/7 and J3/4 are 6-nt and 3-nt long, respectively. This situation is specific to DiGIR2 and is closer to introns that contain a P2/P2.1 extension like the *Tetrahymena* IC1 ribozyme. The group IC3 introns, like the *Azoarcus* ribozyme, contain a simple P2 hairpin. In these, P3 is 6-bp long,

J8/7 and J3/4 are 6-nt and 4-nt long, respectively. In accordance with the *Tetrahymena* ribozyme crystal structure, a triple interaction was built in the DiGIR2 model by docking the Watson-Crick edge of U224 in the narrow groove of the second base-pair of P3, A77-U194. Concerning J3/4, the 5′ residue of J3/4 in the *Azoarcus* ribozyme falls in place with U83 from DiGIR2 involved in the terminal base-pair of P3 with A189. Thus, the additional base-pair of P3 in group IE introns compensates a shorter J3/4 as compared to group IC3 ribozymes.

```
                           P5′           P5″          L9
Dir.S956-1         5′ UCUG..(L5)..CAGA.(core).GCGA. 3′
Dsa.S956           5′ UCUC........CAGA........GUGA. 3′
Dfa.L1975          5′ CCUC........GAGG........GUGA. 3′
Dni.L1975          5′ UCUC........GAGA........GCGA. 3′
Pvi.S956           5′ CCUG........CAGG........GCGA. 3′
Dle.S956           5′ UCUG........CAGA........GUGA. 3′
Dni.S956           5′ ACCC........GGGU........GUAA. 3′
Mob.S956           5′ AGGG........CCUU........GUAA. 3′
Hab.S956           5′ CCCU........AGGG........GUAA. 3′
Csi.S956           5′ GC-U........AGGU........GUAA. 3′
Lsa.S956           5′ GGCC........GGUU........GUAA. 3′
Lov.S956           5′ GCCU........AGGU........GUAA. 3′
Skn.S956           5′ GCUA........UGGG........GAAA. 3′
Cps.S956           5′ GCCU........AGGU........GUAA. 3′
Dme.S956           5′ UCUG........CAGA........GUGA. 3′
```

Figure S3. Sequence covariation between the P5 receptor and L9 sequence. Intron nomenclature is according to [8]. Abbreviated names stand for *Didymium iridis* (Dir), *Diderma saundersii* (Dsa), *Physarum bivalve* (Pbi), *Diderma fallax* (Dfa), *Diderma niveum* (Dni), *Physarum viresens* (Pvi), *Diachea leucopodia* (Dle), *Cribraria cancellata* (Cca), *Macbrideola oblonga* (Mob), *Hermitrichia abietina* (Hab), *Comatricha sinuatocolumellata* (Csi), *Lamproderma sauteri* (Lsa), *Lamproderma ovoideum* (Lov), *Spizellomyces kniepii* (Skn), *Comatricha pseudoaplina* (Cps), *Diderma meyeriae* (Dme).

After building the L-IVS core, the regions non-homologous to the *Azoarcus* ribozyme, namely P2–P2.1 and the P9.1–P9.2 extensions characteristic of group IE introns [9] were modelled step by step in order to form the P13 pseudoknot (unpublished data). The model of the full intron of group IC1 from *Tetrahymena* [10] which displays a P13 element also formed by the interaction between the loops of P2.1 and P9.1 was used as a starting point to elucidate the conformation of these appendages in DiGIR2 (Figure S5a,b). Accordingly, the P2 and P2.1 elements were stacked head to head to form a rod roughly orthogonal to the P1 helix. The P2.1 helix was oriented towards the P3–P9 side whereas P2 was directed towards the P4–P6 domain. On the opposite side of the ribozyme, the P9 domain consists in a four-way junction (4 WJ) composed by P9a, P9b, P9.1 and P9.2 that extends the 2-bp P9.0 element stacked onto P7 that allows the ribozyme 3′-residue ωG to be accommodated in the G-binding pocket. In order to allow L9b to interact with P5, P9a had to be stacked with P9b. Furthermore, P9.2 was stacked with P9.1 to allow the latter to lie along P7/P3 and interact with L2.1 to finally form the P13 pseudoknot. The conformation of the elements encompassing P2/P2.1 and the P9 insertion is moreover supported by the observation that V1 cleavages are only observed on the P13 strand belonging to P9.1 which is indeed exposed to the solvent in the model whereas the opposite strand is buried.

The P5a appendage in DiGIR2 is much shorter than in the *Tetrahymena* ribozyme [7] and about the same size as in the *Twort* ribozyme [11]. In the crystal structure of the former, the P4–P6 domain is entirely visible in the density map and adopts the same overall fold as the independent domain [12] with the tetraloop L5b interlocked with its receptor located at the junction between P6a and P6b. In the latter, the P5a appendage is not visible in the crystal structure beyond the P5 receptor of the L9 loop. Chemical and enzymatic modifications in DiGIR2 show that the P5a extension folds as a hairpin with residues from the L5a loop and from the internal loop connecting to P5 extensively accessible to Watson-Crick probes. Thus, P5a appears to point into the solvent in an undefined direction rather than folding back to make specific contacts with other regions of the P4–P6 domain. This conclusion is supported by sequences of ribozymes phylogenetically related to DiGIR2 showing

that P5 is often extended by Watson-Crick pairs or by motifs unable to kink the helix, such as asymmetrical bulges or C-loops [13].

The present study represents the first whole atom modelling of a group IE intron ribozyme. Previously, secondary structures and cylinder models of three subgroups of group IE introns have been proposed [9]. In addition, the group IE intron from *Candida* has been modelled based on Fe(II)-EDTA and T1 cleavage patterns [14]. Our experimental data and modelling generally conform to the previously proposed models [9,15]. In particular, the characteristic peripheral elements P2.1, P9.1 and P9.2 and the long-range interaction P13 were all incorporated into the model. In addition, our model contributes important new structural features each specific to the splicing or full-length circularization pathway following the observation of two distinct chemical and enzymatic probing patterns.

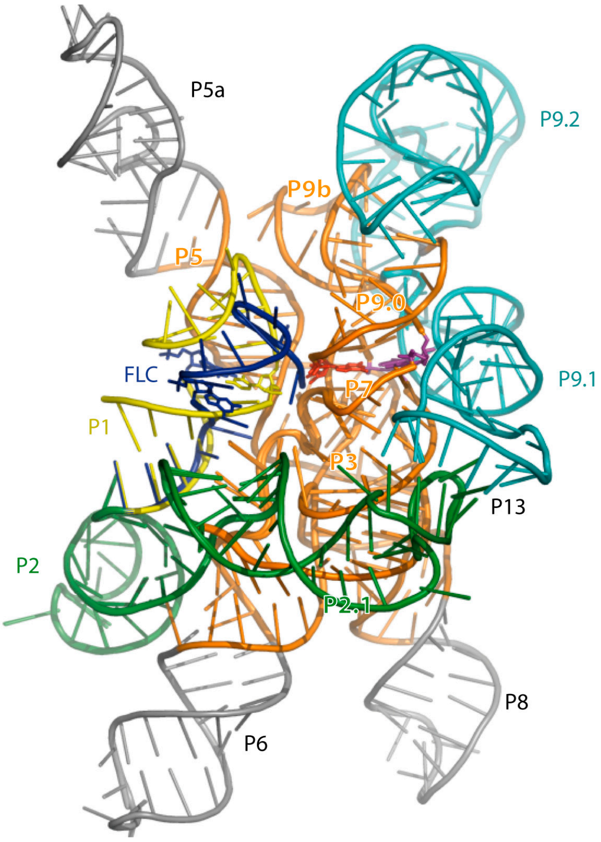

Figure S4. The FLC and L-IVS DiGIR2 ribozyme 3D model overlay. Ribbon nucleotides of the two models were provided by structural homology to the *Azoarcus* group I ribozyme. The structural homologous part to the *Azoarcus* group I ribozyme are depicted in orange (P3, P4, P5, P7, P9b). Nucleotides from the P2/P2.1 and P9.1/P9.2 appendages are depicted in green and cyan, respectively, while the nucleotide extensions of P5a, P6, and P8 specific to DiGIR2 are light gray. The remains of P1 present in the L-IVS are depicted in yellow, while the circle junction of the FLC is materialized in purple. This overlay shows how the loss of the 5′ exon promotes opening of P1 due to the lack of stabilization. Further, the formation of the FLC circle junction leads to a rearrangement of the nucleotides in the region. The loop L9.2 interacts with the J9.0/9a junction promotes and stabilizes the circle junction. The internal loop in P9.1 stabilizes P7 in both L-IVS and FLC molecules.

References

1. Vader, A.; Nielsen, H.; Johansen, S. In vivo expression of the nucleolar group I intron-encoded I-dirI homing endonuclease involves the removal of a spliceosomal intron. *EMBO J.* **1999**, *18*, 1003–1013.
2. Murphy, F.L.; Cech, T.R. An independently folding domain of RNA tertiary structure within the Tetrahymena ribozyme. *Biochemistry* **1993**, *32*, 5291–5300.

3. Haugen, P.; Andreassen, M.; Birgisdottir, A.B.; Johansen, S. Hydrolytic cleavage by a group I intron ribozyme is dependent on RNA structures not important for splicing. *Eur. J. Biochem.* **2004**, *271*, 1015–1024.
4. Adams, P.L.; Stahley, M.R.; Gill, M.L.; Kosek, A.B.; Wang, J.; Strobel, S.A. Crystal structure of a group I intron splicing intermediate. *RNA* **2004**, *10*, 1867–1887.
5. Adams, P.L.; Stahley, M.R.; Kosek, A.B.; Wang, J.; Strobel, S.A. Crystal structure of a self-splicing group I intron with both exons. *Nature* **2004**, *430*, 45–50.
6. Costa, M.; Michel, F. Rules for RNA recognition of GNRA tetraloops deduced by in vitro selection: Comparison with in vivo evolution. *EMBO J.* **1997**, *16*, 3289–3302.
7. Guo, F.; Gooding, A.R.; Cech, T.R. Structure of the Tetrahymena ribozyme: Base triple sandwich and metal ion at the active site. *Mol. Cell* **2004**, *16*, 351–362.
8. Johansen, S.; Haugen, P. A new nomenclature of group I introns in ribosomal DNA. *RNA* **2001**, *7*, 935–936.
9. Li, Z.; Zhang, Y. Predicting the secondary structures and tertiary interactions of 211 group I introns in IE subgroup. *Nucleic Acids Res.* **2005**, *33*, 2118–2128.
10. Lehnert, V.; Jaeger, L.; Michel, F.; Westhof, E. New loop-loop tertiary interactions in self-splicing introns of subgroup IC and ID: A complete 3D model of the Tetrahymena thermophila ribozyme. *Chem. Biol.* **1996**, *3*, 993–1009.
11. Golden, B.L.; Kim, H.; Chase, E. Crystal structure of a phage Twort group I ribozyme-product complex. *Nat. Struct. Mol. Biol.* **2005**, *12*, 82–89.
12. Cate, J.H.; Gooding, A.R.; Podell, E.; Zhou, K.; Golden, B.L.; Kundrot, C.E.; Cech, T.R.; Doudna, J.A. Crystal structure of a group I ribozyme domain: Principles of RNA packing. *Science* **1996**, *273*, 1678–1685.
13. Lescoute, A.; Leontis, N.B.; Massire, C.; Westhof, E. Recurrent structural RNA motifs, Isostericity Matrices and sequence alignments. *Nucleic Acids Res.* **2005**, *33*, 2395–2409.
14. Xiao, M.; Li, T.; Yuan, X.; Shang, Y.; Wang, F.; Chen, S.; Zhang, Y. A peripheral element assembles the compact core structure essential for group I intron self-splicing. *Nucleic Acids Res.* **2005**, *33*, 4602–4611.
15. Suh, S.O.; Jones, K.G.; Blackwell, M. A Group I intron in the nuclear small subunit rRNA gene of Cryptendoxyla hypophloia, an ascomycetous fungus: Evidence for a new major class of Group I introns. *J. Mol. Evol.* **1999**, *48*, 493–500.
